# Osteosarcoma Exosome Priming of Primary Human Lung Fibroblasts Induces an Immune Modulatory and Protumorigenic Phenotype

**DOI:** 10.1158/2767-9764.CRC-24-0371

**Published:** 2025-04-11

**Authors:** Eric P. Palmer, Kathryn E. Cronise, Laurel A. Haines, Sunetra Das, Aaron Offermann, Carina P. Easton, Daniel P. Regan

**Affiliations:** 1 Flint Animal Cancer Center, College of Veterinary Medicine and Biomedical Sciences, Colorado State University, Fort Collins, Colorado.; 2 Department of Microbiology, Immunology, and Pathology, College of Veterinary Medicine and Biomedical Sciences, Colorado State University, Fort Collins, Colorado.; 3 Department of Clinical Sciences, College of Veterinary Medicine and Biomedical Sciences, Colorado State University, Fort Collins, Colorado.

## Abstract

Osteosarcoma (OS) is the most common malignant bone cancer. Thirty to forty percent of all patients with OS develop tumor recurrence, almost exclusively in the form of lung metastasis, which is associated with a dismal 20% 5-year survival rate. Cancer-associated fibroblasts are a critical cell type within the lung tumor microenvironment that promote immune suppression, drug resistance, and tumor cell survival. Prior work shows tumor cells can co-opt fibroblasts to a protumorigenic phenotype via exosome-mediated intercellular communication. Currently, the mechanisms by which OS exosomes modulate resident lung fibroblast (LF) function have not been evaluated. To investigate this, we isolated exosomes from a panel of six OS cell lines. We assessed the uptake and response of human donor–derived primary LFs (*n* = 4) to OS exosome treatment *in vitro* via flow cytometry, confocal fluorescent microscopy, proliferation assays, phosphokinase array, multiplex cytokine analysis, and RNA sequencing. We observed that LFs efficiently take up OS exosomes, which are associated with the induction of MAPK pathway activation, fibroblast proliferation, and significantly enhanced secretion of IL-6, CXCL8, and CCL2 compared with untreated LFs. RNA sequencing of exosome-treated LFs confirmed these responses and revealed significant enrichment of pathways related to cytokine secretion, proliferation, immune cell chemotaxis, migration, proinflammatory, and profibrotic mediators. Finally, in an exosome-educated LF–OS coculture model, exosome-educated LFs conferred significantly increased OS cell survival and proliferation compared with untreated fibroblasts. Together, these data suggest that the OS-derived exosomes can induce a hallmark cancer-associated fibroblast–like inflammatory phenotype in LFs, providing valuable insights into mechanisms that may promote recurrent OS lung metastasis.

**Significance:**

These findings provide a critical first step in characterizing the capacity of OS-derived exosomes to reprogram primary LFs toward a tumor-promoting inflammatory phenotype *in vitro*, offering novel molecular targets for the modulation of fibroblasts in the lung microenvironment as potential therapeutic strategies to prevent OS metastasis.

## Introduction

Osteosarcoma (OS) is a highly malignant primary bone tumor characterized by its aggressive behavior, predilection for occurrence in children and young adults, and recurrent disease that presents almost exclusively in the form of lung metastasis (1–3). Despite the discovery of intrinsic molecular drivers of OS progression and promising preclinical studies demonstrating the efficacy of immune checkpoint inhibitors and molecular targeted therapies, clinical outcomes for patients with OS remain unchanged, and patients continue to receive the same multidrug chemotherapy protocol of methotrexate, adriamycin, and cisplatin developed over three decades ago (4–11). The disconnect between preclinical models of metastasis and clinical efficacy highlights a failure of current models to fully recapitulate the complexity of key cellular players and signaling mechanisms within the tumor microenvironment (TME), their role in extrinsically driving disease progression and therapy resistance, and the urgent need to develop new models that accelerate progress toward novel therapeutic interventions (12).

Among the intricate network of factors that contribute to OS progression, the role of extracellular vesicles, particularly exosomes, has begun to emerge as a key signaling mechanism (13). Exosomes are nano-sized vesicles secreted by various cell types, including cancer cells, and have garnered considerable attention for their ability to mediate intercellular communication through the transfer of bioactive molecules such as proteins, lipids, and nucleic acids (14, 15). Furthermore, evidence suggests that cancer-derived exosomes are implicated in a myriad of processes within the local TME that promote tumor growth and metastasis, immune evasion, angiogenesis, and epithelial-to-mesenchymal transition (EMT; refs. 16–21). Importantly, exosomes have been shown to traffic to site-specific organs and mediate the remote priming of resident cells to promote the formation of a specialized microenvironment enhancing the survival, growth, and colonization of incoming, circulating cancer cells, a key process in the conditioning of the premetastatic niche (PMN; refs. 22–25).

Lung fibroblasts (LFs), traditionally viewed as an essential component of the pulmonary stroma for their multifaceted role in maintaining tissue homeostasis and response to injury, are now recognized as regulators of various pathologic processes, including inflammation, fibrosis, and cancer (26–29). Evidence suggests fibroblasts can undergo dynamic alterations in response to signaling from the primary tumor. Within this context, LFs have been identified as a key cellular target of tumor-secreted exosomes, leading to their co-optation as active participants in PMN formation through cellular and molecular events, such as extracellular matrix remodeling, secretion of growth factors and chemokines, and immunomodulation (27, 30, 31). These “activated” or cancer-associated fibroblasts (CAF) comprise a diverse array of functionally distinct subtypes, each of which exerts a profound influence over the architectural and biochemical landscape of the lung PMN (32).

Thus, although mounting evidence has implicated tumor-derived exosomes and nonmalignant resident cells as effectors of organ-specific metastasis in multiple cancers such as melanoma, breast, liver, and pancreatic (16, 22, 23), the cellular and molecular mechanisms governing OS’s striking predilection for metastasis to the lung remain to be fully elucidated. This is a key knowledge gap in OS metastasis biology that, if addressed, could identify novel molecular targets for host-directed antimetastatic therapies or biomarkers for identifying those patients with OS at high risk for metastasis. Therefore, we investigated the role OS-derived exosome signaling plays in promoting the transition of primary donor-derived human LFs toward a tumor-promoting phenotype. We show that OS-derived exosomes are efficiently taken up by LFs *in vitro*, inducing enhanced fibroblast proliferation, MAPK signal transduction, and secretion of cytokines and chemokines previously implicated in OS lung metastasis, including IL-6, CXCL8, and CCL2. Additionally, transcriptomic analysis of LF response to OS exosome priming demonstrated significant enrichment of genes associated with a protumorigenic secretory profile and enhanced proliferation, as well as genes associated with the promotion of adhesion, extracellular matrix (ECM) remodeling, and angiogenesis. Furthermore, we show that OS exosome activation elicits protumorigenic functions in LFs, including monocyte recruitment and enhanced survival of OS cells in a fibroblast–OS cell coculture model. These data provide a mechanistic basis for potential molecular targets that may be effective in modulating the protumorigenic functions of fibroblasts in the lung microenvironment as a therapeutic strategy for OS metastasis.

## Materials and Methods

### Cell lines

Human OS cancer cell lines (143b, HOS, MG63.0, MG63.2, U2OS, and SAOS-2) and other cancer cell lines (Malme-3M, TCCSUP, and T84) were purchased from the ATCC. Breast cancer cell lines (MDA-MB-231 and MDA-MB-468) were purchased from the University of Colorado Cancer Center Tissue Culture Shared Resource. Normal human lung fibroblasts (NHLF) from *n* = 4 individual donors were purchased from Lonza Inc. Additional information about LF donor age and sex is provided in Supplementary Table S1. All tumor cell lines were validated by short tandem repeat analysis, and all cell lines were routinely tested for mycoplasma contamination using a commercially available assay (MycoAlert from Lonza Inc.). Tumor cells were maintained in DMEM (Gibco) or RPMI (MB231 and MB468) supplemented with 10% FBS (Atlas Biologicals), penicillin (100 U/mL), and streptomycin (100 μg/mL; all from Gibco). NHLFs were maintained in FBM-2 (Lonza Inc.) supplemented with insulin, human fibrblast growth factor, gentamicin, and 2% FBS (complete FGM). Cells were grown sterilely on standard plastic tissue culture flasks (CELLTREAT) under standard conditions of 37°C, 5% CO_2_, and humidified air. To transduce OS cell lines using IncuCyte Nuclight rapid red cell labeling lentivirus constructs (Sartorius, cat. # 4771), 5,000 OS cells were plated (15%–35% confluent) in flat-bottom 96-well plates in complete DMEM for 24 hours. Media were removed and replaced with complete DMEM containing NucLight lentivirus at a multiplicity of infection of 3 (8 µg/mL) for 72 hours while monitoring for red fluorescent protein (RFP) expression using the IncuCyte S3 live-cell imaging system (Sartorius). Subsequently, RFP-labeled cells were selected for using 1 µg/mL puromycin in complete DMEM while being monitored for expression selection by IncuCyte over the course of 3 days.

### Isolation of exosomes

Cancer cell lines were seeded in five-deck multiflasks (Corning Falcon, cat. # 353144) at ∼20% confluence (roughly one million cells) in their respective media containing 10% FBS for 24 hours. Subsequently, the media were removed and replaced with their respective base media containing only 2% exosome-depleted FBS for 3 days before harvesting this tumor conditioned media (CM) for exosome isolation. FBS was depleted of exosomes by differential ultracentrifugation [Optima MAX-XP ultracentrifuge (cat. # 393315) and MLA-55 rotor (cat. # 393203), Beckman Coulter] at 120,000 × *g* for 3 hours at 4°C and was confirmed to be depleted of exogenous exosomes by qNano Gold (Izon Science) tunable resistive pulse sensing particle analysis, as described below (33). To isolate tumor-derived exosomes, tumor CM was first centrifuged at 3,000 × *g* for 10 minutes at 4°C to remove cellular debris and then subjected to ultrafiltration using Amicon ultra centrifugal filters (Centricon Plus-70) with a 100 kDa molecular weight cutoff to concentrate extracellular vesicles (EVs). To prepare the Centricon Plus-70 centrifugal filter (Sigma, cat. # UFC710008) for the concentration of tumor CM, 35 mL of PBS was loaded and centrifuged at 3,500 × *g* and 4°C for 10 minutes to remove glycerol from the filter. After discarding the PBS flow-through, 70 mL of prepared tumor CM was loaded onto the filter and centrifuged at 3,500 × *g* for 45 minutes at 4°C. The flow-through was discarded, followed by the addition of 35 mL of PBS to the filter and centrifuged at 3,500 × *g* and 4°C for 15 minutes to wash the column. Flow-through was once again discarded, and the filters were flipped upside down, placed on the collection cup, and centrifuged at 1,000 × *g* and 4°C for 2 minutes. This concentrated ultrafiltrate of tumor CM collected from the centrifugal filters was then subjected to size-exclusion chromatography to isolate a pure population of tumor-derived exosomes using qEVoriginal 35 nm size-exclusion chromatography columns and an automatic fraction collector (Izon Science). Samples were eluted with 1X PBS, and the void volume and all ten 0.5 mL eluted fractions were collected and subjected to nanoparticle tracking and micro BCA protein analyses for quantification of extracellular vesicle and protein concentrations. Fractions 1 to 3, identified as the particle-rich, protein-poor fractions, as reported in the QeV Original User Manual, were pooled and sterile filtered using 45 µm polyethersulfone membrane syringe filters (Merck Millipore Ltd., cat. # SLGPR33S) prior to further characterization for exosome purity, as described below. Chromatography columns were reused no more than five times per the manufacturer’s recommendation and were washed with PBS three times between uses.

### Exosome characterization

For transmission electron microscopy (TEM), exosome pellets were loaded onto formvar-/carbon-coated TEM grids, fixed with 2% paraformaldehyde, and stained with 1% aqueous uranyl acetate. The samples were then examined using a TEM (JEM-1400, JEOL). For nanoparticle tracking analysis, particle concentrates were diluted 1:10 in 3× PBS and then examined using a tunable resistive pulse sensing instrument, qNano Gold (Izon Science). A nanopore 150 at 47 nm stretch, 24 V at approximately 100 nA was applied and measured against standard particles of 100 nm in diameter at 1.3 × 10^–13^ particles per milliliter.

For Western blot analysis of canonical exosome surface proteins, 200 μL of exosome concentrates at 1 × 10^10^ particles per milliliter were ultracentrifuged at 100,000 × *g* on an ultracentrifuge (MAX-XP, Beckman Coulter) for 1 hour. Pellets were lysed using M-PER lysis buffer (Thermo Fisher Scientific, cat. # 78501) supplemented with protease and phosphatase inhibitor mini tablets (Pierce, cat. # A32961) prior to the addition of 4× Laemmli loading buffer with 10% beta-mercaptoethanol followed by boiling at 95°C for 5 minutes. Proteins were run on an SDS–PAGE gel and transferred to a polyvinylidene difluoride membrane. Membranes were blocked with 5% BSA in TBS with 0.1% Tween 20 and then incubated independently with the following primary antibodies: rabbit anti-CD9 (D801A, Cell Signaling Technology) and rabbit anti-CD81 (D3N2D, Cell Signaling Technology). For flow cytometry of exosome markers, 1 × 10^10^ exosomes per milliliter were ultracentrifuged at 100 × *g* for 1 hour; the pellet was resuspended in 100 μL of FACS buffer containing mouse anti-human CD9-APC (MEM-61, Invitrogen), CD81-FITC (M38, Life Technologies), and CD63-PacBlue (H5C6, BioLegend) antibodies for 15 minutes before quenching with 1 mL of FACS buffer. Two washing steps of ultracentrifugation with resuspension in 1 mL of FACS buffer were performed, followed by a final resuspension in 150 μL of FACS buffer prior to flow cytometric analysis on a Cytek Aurora four-laser spectral flow cytometer.

### Assessment of tumor exosome uptake by LFs

To evaluate OS cell line–derived exosome uptake by NHLFs *in vitro*, 1 × 10^10^ exosomes per milliliter were ultracentrifuged at 100,000 × *g* for 1 hour to pellet the exosomes, further concentrating them and allowing for the removal of PBS supernatant. Pellets were resuspended in 100 μL of diluent C and then labeled with the lipophilic membrane dye PKH26 (Sigma, PKH26GL) at 1 µmol/L for 5 minutes prior to the addition of 200 μL of water. Labeled exosomes or equimolar dye–only vehicle controls were centrifuged again at 100,000 × *g* for 1 hour to pellet the exosomes and separate them from unwanted, unincorporated dye particles. Supernatants containing unincorporated dye were removed, and the labeled exosome pellets or dye-only controls were resuspended in 100 μL PBS and 800 μL serum-free DMEM (sf-DMEM) for use in the following NHLF exosome uptake experiments.

NHLFs were plated either in flat-bottom 96-well plates at 20,000 cells per well for flow cytometry or in chamber slides (Thermo Fisher Scientific, cat. # 177445) at 5,000 cells per well (fluorescence microscopy) in complete FGM and allowed to adhere overnight. The next day, media were removed and replaced with 200 µL of serum-free media containing PKH-26–labeled OS-derived exosomes at 1 × 10^9^ exomes per milliliter and incubated at 37°C for 4 hours. For flow cytometric analysis of OS exosome uptake, NHLF cells were washed with PBS, trypsinized, and then treated with Zombie NIR (BioLegend, cat. # 423105) viability dye in PBS containing 5 µmol/L ethylenediaminetetraacetic acid for 15 minutes. Cells were then washed twice in FACS buffer prior to being analyzed by a Cytek Aurora four-laser flow cytometer. For confocal microscopy of NHLF after the 4-hour exosome incubation, cells were washed twice with PBS and then fixed using 4% paraformaldehyde (Thermo Fisher Scientific, cat. # J19943.K2) diluted 1:1 with PBS for 10 minutes prior to imaging. Fixed cells were stained with ActinGreen (Invitrogen, cat. # R37110) following recommended protocols; then chamber slides were coverslipped with DAPI containing ProLong diamond antifade mountant (Invitrogen) and allowed to dry overnight prior to imaging. Confocal fluorescence imaging was performed using an Olympus IX83 spinning disc microscope, Hamamatsu ORCA-R2 digital camera, and CellSens software (Olympus, version 4.2), and both high-magnification single-field and low-magnification whole-slide images were captured using standardized exposure times across vehicle control and PKH26 exosome–treated NHLF slides.

### Phosphokinase analysis of LFs

Kinase activation in exosome-treated LFs was assessed using the proteome profiler human phosphokinase array kit (R&D Systems, cat. # ARY003). A total of 300,000 NHLFs (*n* = 2 biologically independent donors, F62, M12; see Supplementary Table S1) were plated in 25 cm^2^ tissue culture flasks for each treatment condition overnight in complete FGM. After 24 hours, the media were replaced with either 5 mL of OS-derived exosomes (143b) at 5 × 10^9^ OS exosomes per milliliter in sf-DMEM or sf-DMEM alone (control). Subsequently, fibroblasts received two additional exosome treatments at 24 and 72 hours, removing 2.5 mL of the CM and replacing it with 2.5 mL of 5 × 10^9^ OS exosomes per milliliter in sf-DMEM or sf-DMEM only for control wells. Four hours after the last exosome treatment, cells were washed with PBS and lysed using the lysis buffer provided in the array kit. Protein concentration was measured using a Pierce BCA assay kit (Thermo Fisher Scientific), and equal amounts of control and exosome-treated LF protein lysates were analyzed by immunoblotting according to the kit manufacturer’s protocol. Membranes were imaged on a ChemiDoc imaging system (Bio-Rad) at equivalent exposure times (5 minutes) to maximize the signal-to-noise ratio. Using ImageJ image analysis software to quantify phosphoprotein abundance, membrane images were analyzed for integrated pixel density. The average signal (pixel density) of the pair of duplicate spots representing each phosphorylated kinase protein was determined, and then an averaged background signal, generated from a clear array of the membrane, was subtracted from each spot. These values were then compared between control and exosome-treated NHLF to determine the relative change in phosphorylated kinase proteins between samples.

Additionally, OS exosome–induced kinase activation in human LFs was quantitatively assessed using MILLIPLEX MAPK/stress-activated protein kinase and Akt/mTOR phosphoprotein magnetic bead–based assays (Sigma-Aldrich, cat. # 48-660MAG, #48-611MAG) and a MAGPIX instrument (Luminex). For these assays, 10,000 NHLFs were plated per well in 96-well flat-bottom plates and allowed to adhere overnight in complete FGM. Twenty-four hours after plating, the media were removed, and NHLFs were treated with either sf-DMEM containing 5 × 10^9^ OS exosomes per milliliter (*N* = 5 OS cell lines) or sf-DMEM alone. OS exosome education was then repeated 72 hours after plating. At 96 hours after plating, NHLFs were washed with 200 µL PBS and lysed using M-PER buffer (Thermo Fisher Scientific) containing a Pierce protease/phosphatase inhibitor cocktail (Thermo Fisher Scientific). Lysates were processed and analyzed for phosphoprotein relative abundance according to the manufacturer’s protocol. Samples were analyzed using the Luminex MAGPIX instrument. Quality control and initial processing of data were performed with Luminex xPONENT software prior to export and further analysis with GraphPad Prism version 10.0.1.

### RNA sequencing analysis of OS exosome–treated primary human donor LFs

NHLFs from a single donor (M48) were plated in triplicate at 100,000 cells/well in 24-well plates in complete FGM and allowed to adhere overnight. Cells were then treated with HOS, 143B, MG63.0, or MG63.2 OS exosomes at 5 × 10^9^ particles per milliliter the next day (∼24 hours after plating) and again at 72 hours after plating. Twenty-four hours following the last exosome treatment, RNA was extracted using a Qiagen RNeasy mini kit. Extracted RNA was sent to Novogene Corp. Ltd. for sequencing. Samples were tested for quality control by the Agilent 2100 Bioanalyzer system and by agarose gel electrophoresis. Libraries were run on a NovaSeq 6000 to generate 150 bp paired-end reads to a target depth of 40 mol/L raw reads per sample. Following quality control (FASTQC version 0.11.8) and adaptor removal (Trimmomatic version 0.39), FASTQ reads were aligned to the human reference genome GRCh38 (Ensembl release 107) using the STAR alignment tool (version 2.7.10a).

Following alignment, the remainder of downstream analyses was performed using R statistical software (version 4.2.3). Unexpressed or lowly expressed genes were filtered out by requiring a minimum count of 10 in at least three samples. Normalization and identification of differentially expressed genes between control and exosome-primed fibroblasts were performed using the DESeq2 package (version 1.38.3), requiring an FDR <0.05 and a fold change cutoff of 2. Differentially expressed genes were analyzed for enrichment of pathways/processes by screening against curated gene sets within the Molecular Signatures Database (Hallmarks, CP:BioCarta, CP:PID, CP:Reactome, CP:WikiPathways, GO:BP, and GO:MF collections) using the “enricher” function within the clusterProfiler (version 4.6.2) package (FDR <0.05). Graphical representations of gene expression analyses were created using ggplot2 (version 3.4.4), EnhancedVolcano (version 1.16.0), and ComplexHeatmap (version 2.14.0).

### Multiplex cytokine analysis of LFs

Multiplex cytokine analysis of control and OS exosome–treated LFs was performed using a human cytokine/chemokine/growth factor 45-Plex Panel (Invitrogen, EPXR450-12171-901). Both male and female donor NHLFs (M29 and F62) were treated with OS exosomes from *n* = 4 cell lines that were sex-matched to the fibroblast donor. Male cell lines were MG63.0 and MG63.2, and female cell lines were 143B and HOS. For exosome treatment, 10,000 NHLFs were plated in triplicate in 96-well plates and allowed to adhere overnight in complete FGM. At 24 and 72 hours after plating, NHLFs were treated with either sf-DMEM alone (control) or OS-derived exosomes at 5 × 10^9^ exosomes/mL in sf-DMEM. Twenty-four hours after the last exosome treatment, CM was harvested, and the supernatant was obtained by centrifugation at 10,000 × *g* to remove cell debris. Cytokine concentration in the NHLF supernatant was then measured according to the kit manufacturer’s protocol. In a second set of independent experiments, 10,000 NHLFs (*n* = 3 donors; F62, M29, and M12) were plated in the same manner and underwent the same tumor-derived exosome treatment protocol as described above. Exosome treatment conditions included OS exosomes (*n* = 5 cell lines), other tumor histotype exosomes (*n* = 5; MB231, MB468, Malme-3M, T84, and TCCSUP), or untreated control (sf-DMEM). CM from these conditions as well as tumor-derived exosome preparations alone (*n* = 10) were analyzed for cytokine secretion using the same kit and protocol as described above. Samples were run on a Luminex MAGPIX instrument. Quality control and initial processing of data were performed with Luminex xPONENT software prior to export, and further analysis was performed with GraphPad Prism version 10.0.1.

### Primary human LF–OS cell coculture experiments

A total of 10,000 NHLFs were plated in triplicate in flat-bottom 96-well plates and allowed to adhere overnight in complete FGM. The next day, media were removed, and NHLFs were then treated with either sf-DMEM alone (vehicle) or sf-DMEM containing OS exosomes (*n* = 4) at 5 × 10^9^ exosomes/mL every 48 hours for three total treatments. Twenty-four hours after the third exosome treatment, NucLight red–labeled OS cells in sf-DMEM were seeded in triplicate at 100 cells/well either alone (control) or in wells containing “uneducated” NHLF or NHLF “educated” with OS exosomes matched to that respective OS cell line. RFP + OS cell survival/proliferation was monitored and quantified using the IncuCyte S3 live-cell imaging system for 7 days. During this 7-day culture period, OS-NHLF coculture wells either received no additional exosome treatment or received three additional OS exosome treatments (5 × 10^9^ exosomes/mL every 48 hours). Nuclear-labeled (RFP) OS cell survival and proliferation were assessed every 24 hours for 7 days using the IncuCyte S3 live-cell imaging system (Sartorius). RFP + OS cells per well were quantified using the IncuCyte Image Analysis software, and OS cell survival/proliferation was quantified as the fold change in RFP + cell count per well by dividing day 7 RFP + OS cell counts by postplating baseline RFP + OS cell counts.

### NHLF cell survival/proliferation assays

A total of 7,500 NHLFs were plated in 96-well plates in triplicate overnight in complete FGM. NHLFs were treated with 1 × 10^9^ exosomes per milliliter (*n* = 4) 24 and 72 hours after plating. At 96 hours after plating, NHLFs were fixed using 2% paraformaldehyde for 10 minutes prior to being fluorescently labeled using NucSpot live-cell nuclear labeling (Biotium) following the manufacturer’s protocols and then imaged using the IncuCyte S3 live-cell imaging system (Sartorius). Cell survival/proliferation was quantified as an endpoint change in RFP + cell count per well.

### Western blot analysis of exosome-treated NHLF

Changes in canonical CAF marker expression by NHLF in response to OS exosome treatment were assessed by Western blot analysis. NHLFs were treated with exosomes as previously described; lysates were generated using M-PER buffer (Thermo Fisher Scientific) supplemented with phosphatase and protease inhibitors (Pierce), and protein concentration was measured by BCA. Ten micrograms of protein was loaded into each well in a 10-well Mini-PROTEAN TGX gel (4% to 20%; Bio-Rad, cat. # 4561094) run in 1× loading buffer (Bio-Rad, cat. # 10026938) at 180 V for 40 minutes on a PowerPac basic power supply (Bio-Rad, cat. # 1645050) prior to transfer to a polyvinylidene difluoride membrane (Bio-Rad, cat. # 1620174) using the Trans-Blot turbo transfer system (Bio-Rad, cat. # 17001915) for 7 minutes. Membranes were blocked for 1 hour in 5% BSA in tris-buffered saline with tween 20 (TBST) prior to overnight incubation at 4°C with anti-human, rabbit fibroblast activation protein, and α-smooth muscle actin (α-SMA) antibodies diluted 1:1,000 in 5% BSA in TBST (Invitrogen, cat. # PA5-32765; Proteintech, cat. # 55135). Membranes were washed three times for 5 minutes each using TBST prior to the application of secondary anti-rabbit horseradish peroxidase-conjugated antibody (Cell Signaling Technology, cat. #7074) diluted to 1:5,000 in 5% BSA in TBST for 1 hour, followed by three additional 5-minute washing steps in TBST. Membranes were imaged using the ChemiDoc XRS+ imaging system (Bio-Rad) with the Clarity Max Western ECL substrate (Bio-Rad, cat. # 170562). Densitometry analysis was performed using ImageJ software, and adjusted band volume was normalized to the loading control (GAPDH).

### Migration assay

NHLFs (M48) were plated in triplicate in 12-well plates at 150,000 cells per well for 24 hours in FGM. Then, the media were removed and replaced with sf-DMEM alone as a vehicle control or with 1 × 10^9^ exosomes per milliliter in sf-DMEM at 0.5 mL with full media replacement per well at 24 and 72 hours after plating. Supernatants were collected, centrifuged at 10,000 × *g* to remove cell debris, and validated for IL-6, CXCL8, and CCL2 concentrations by ELISA. One hundred and fifty microliter of exosome-treated fibroblast supernatants, sf-DMEM as a negative control, 10% serum complete DMEM, and sf-DMEM containing 1 ng/mL recombinant CCL2 (PeproTech, cat. # 300-04) as positive controls were loaded into the bottom chamber of a 5 µm HTS Transwell 96-well support (Corning, cat. # 3387). Prior to this, THP-1 cells (ATCC, cat. # TIB-202) were grown in complete serum RPMI until reaching the desired concentration. Cells were counted and plated in the upper chamber of the Transwell support in 50 µL at 50,000 cells per well for 4 hours followed by image analysis using confocal microscopy.

### Statistical analyses

Samples were not randomly assigned to experimental groups, and experimenters were not blind to group assignment and outcome assessment. For the comparison of mean values between three or more groups, a one-way ANOVA with Tukey’s posttest was performed. For the comparison of means between two groups or the comparison of repeated measures between two groups, a two-tailed, unpaired Student *t* test or Wilcoxon matched-pairs signed-rank test was used, respectively. All statistical analyses were performed using GraphPad Prism software, version 10. Unless otherwise specified, all data are representative of at least three independent experiments, and all results are expressed as the mean ± SD, with each dot representing individual measurements.

### Data availability

All data generated in this study are available within the article and its supplementary data files and are available from the corresponding author upon request. Raw RNA sequencing data from this study are publicly available for download from the Sequence Read Archive under BioProject PRJNA1231444.

## Results

### Isolation and characterization of OS cell–derived exosomes

**Figure 1:**
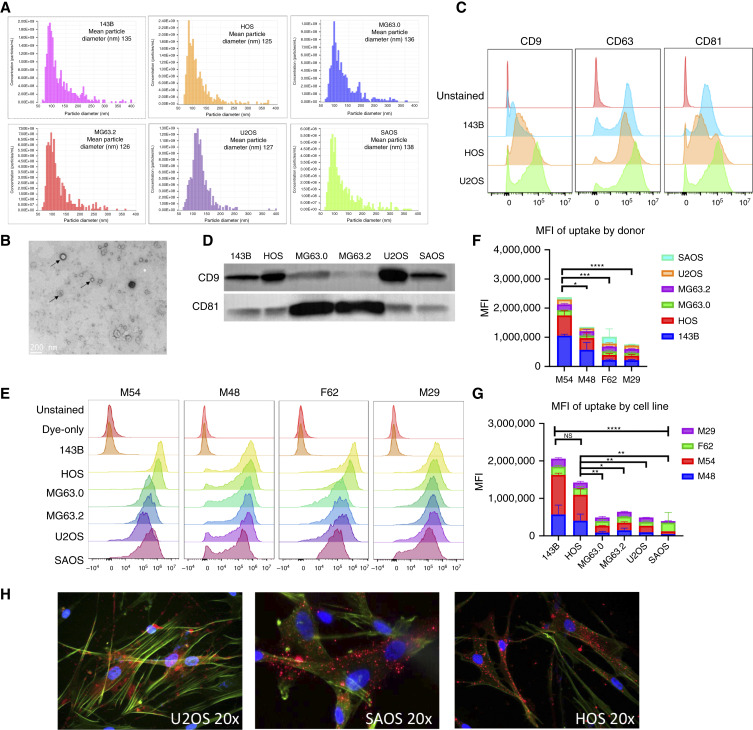
Characterization of isolated OS-derived exosomes and quantification of OS exosome uptake in NHLF. **A,** Tunable resistive pulse sensing (qNano) analysis of exosomes isolated from OS cell lines (*n* = 6) represents median and mode particle diameter and particle counts per milliliter. **B,** Transmission electron micrograph of 2% uranyl acetate negative staining of 143B exosomes captured on carbon-coated microgrids. Scale bar, 200 nm. **C,** Flow cytometry analysis of exosome markers CD9, CD63, and CD81 on OS cell lines (*n* = 3). **D,** Western blot analysis of exosome markers CD9 and CD81 on OS-derived exosomes (*n* = 6). **E,** Flow cytometry analysis of internalized fluorescently labeled (PKH26) exosomes from six OS cell lines, dye-only control, and 143B unstained control across four donor-derived NHLF cell lines. **F,** Mean fluorescent intensity (MFI) of PKH26-OS exosome uptake (*n* = 6) separated by donor NHLF (*n* = 4). **G,** MFI of PKH26-OS exosome uptake separated by OS cell line. Data and results gathered from three technical replicates. **H,** Fluorescent confocal microscopy images of PKH26–OS exosomes (*n* = 3) internalized by actin (green) and DAPI (blue) counterstained F62, donor-derived NHLF. Two-way ANOVA and Tukey’s multiple comparisons test were used. *, <0.05; **, <0.01; ***, <0.001; ****, <0.0001. NS, not statistically significant.

To first confirm our ability to effectively isolate a pure population of exosomes from human OS cell lines, we characterized exosome production from the paired isogenic high- and low-metastatic cell lines 143B/HOS and MG63.2/MG63.0, respectively, as well as U2OS and SAOS-2 cells. Extracellular vesicles enriched from OS cell CM by ultrafiltration and size-exclusion chromatography were of high concentration and of the expected size range for exosomes based on particle analysis by tunable resistive pulse sensing, with a mean particle diameter ranging from 125 to 138 nmol/L and mode particle diameter ranging from 86 to 116 nmol/L (Fig. 1A). Additionally, 143B and SAOS-2 exosomes were imaged using TEM, which also demonstrated the expected size and sphere-shaped morphology of exosomes (Fig. 1B). Moreover, flow cytometric analysis of these particles demonstrated high expression of the putative exosome markers CD9, CD63, and CD81 (Fig. 1C), which was also confirmed by Western blot analysis (Fig. 1D).

### Primary human donor–derived NHLFs efficiently internalize OS exosomes *in vitro*

To determine if NHLFs are capable of internalizing OS-derived exosomes *in vitro*, NHLFs from both male and female donors (*n* = 4 independent donors, Supplementary Table S1) were treated with equal numbers of PKH26-labeled OS exosomes from *n* = 6 OS cell lines for 4 hours prior to flow cytometric analysis. PKH26-labeled exosomes from all six OS cell lines were efficiently taken up by all four independent donor NHLF cell lines *in vitro*, with more than 90% uptake compared with the unstained exosomes and dye-only controls (Fig. 1E–G; Supplementary Table S2). Evaluation of PKH26^+^ exosome geometric mean fluorescent intensity across fibroblast donors and OS cell lines demonstrated differences in exosome uptake according to fibroblast donor and OS cell line, with the high-metastatic cell line 143b demonstrating the greatest mean uptake of exosomes across all donors, and M54 NHLF internalizing more PKH26^+^ exosomes compared with other donors (Fig. 1F and G). Confocal fluorescent microscopy of NHLF (donor F62) treated for 4 hours with PKH26-labeled exosomes also confirmed intracellular uptake of OS-derived exosomes (Fig. 1H). These data demonstrate that primary human LFs are capable of robust internalization of OS exosomes and that this exosome uptake is conserved across a panel of OS cell lines and multiple independent male and female donor LFs.

### RNA sequencing of OS exosome–treated fibroblasts reveals gene signatures associated with hallmark CAF phenotypes

**Figure 2:**
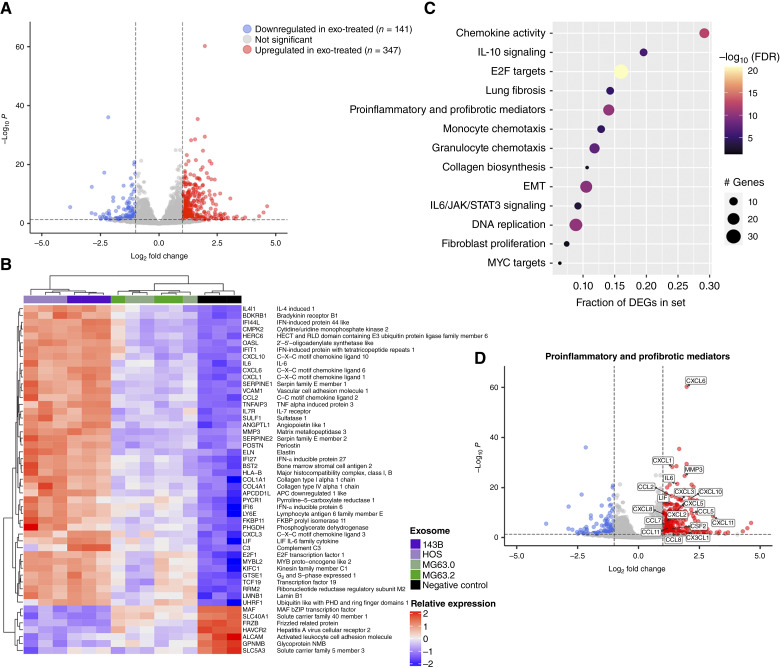
OS-derived exosomes induce differential expression of genes associated with hallmark CAF phenotypes. **A,** Volcano plot of differentially expressed genes (DEGs) in NHLF in response to OS exosome treatment (*n* = 4 human OS cell lines). Three hundred and forty-seven upregulated genes (red) and 141 downregulated genes (blue) at adjusted *P* value < 0.05 and fold-change cutoff of 2. **B,** Heatmap of the 50 most significant DEGs comparing OS exosome treatment (143b, HOS, MG63.0, MG63.2) and untreated control. **C,** Dot plot of selected enriched pathways/processes from the cluster profiler gene enrichment tool. **D,** Volcano plot of DEGs within the proinflammatory and profibrotic mediators’ gene set.

Given the efficient internalization of OS-derived exosomes by LFs, we sought to identify transcriptomic responses indicative of possible exosome–mediated phenotypic or functional changes in LFs. RNA sequencing of LFs in response to OS-derived exosome treatment revealed 488 significantly differentially expressed genes, with 347 upregulated and 141 downregulated genes (Supplementary Table S3; Fig. 2A). Of the top 50 differentially expressed genes (Fig. 2B), many were associated with hallmark CAF phenotypes, including upregulation of cytokine and chemokine expression (*CXCL-1, CXCL-3, CXCL-6, CXCL-10, IL-6, CCL2*), extracellular matrix remodeling (*MMP3*, periostin, elastin, collagens type 1, collagens type 4), angiogenesis (angiopoietin like 1, bradykinin receptor B1), and proliferation (*E2F1, GTSE1*). Pathway analysis of differentially expressed genes in response to exosome priming revealed enrichment of gene sets related to previously documented biologically relevant CAF subsets, including inflammatory CAFs (iCAF) and myofibroblastic CAFs (myCAF). Enriched gene sets included pathways involved in cytokine and chemokine activity, immune suppression, myeloid cell chemotaxis, as well as proliferation, EMT, and collagen production/fibrosis (Supplementary Table S4; Fig. 2C and D). Furthermore, genes enriched in the gene set “proinflammatory and profibrotic mediators” (Fig. 2D) consist of the upregulation of molecules previously implicated in OS lung metastasis, such as IL-6, CCL2, and CXCL8 (34), or reported to be upregulated in metastasis-associated fibroblasts (MAF) in other cancers (35). Together, these transcriptional changes suggest that OS exosomes can promote the rapid transition of normal LFs into an MAF phenotype.

### OS exosomes promote proliferation and differential kinase activation in LFs

**Figure 3:**
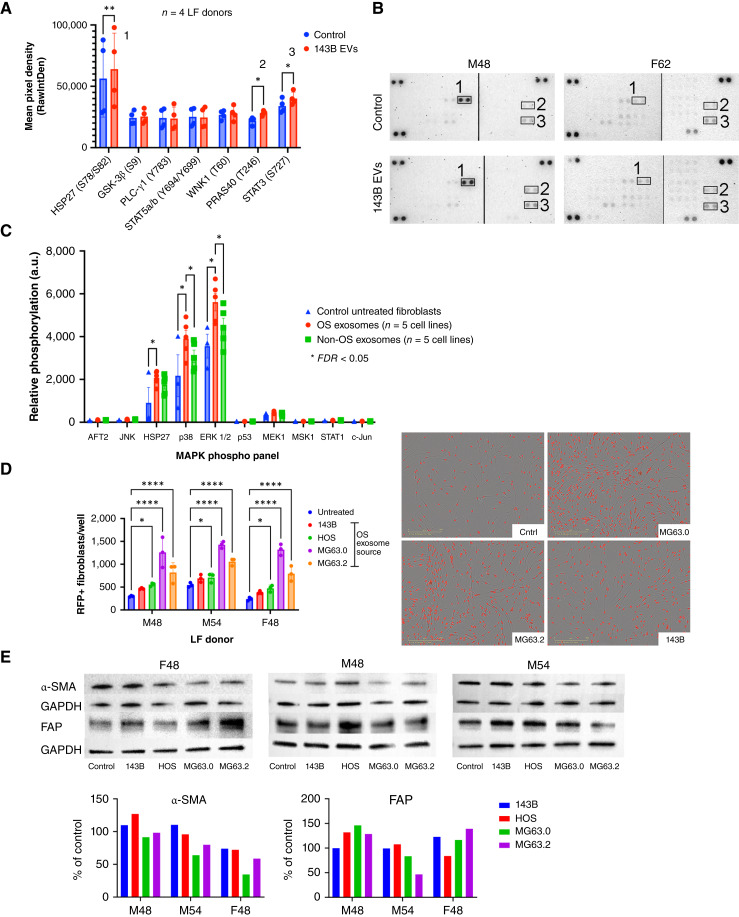
OS-derived exosomes induce signal transduction pathways and enhance proliferation but do not induce the expression of canonical CAF markers in NHLF *in vitro*. **A,** Bar graph of normalized fold-change integrated intensity from a human phosphoprotein array depicting the most upregulated phosphoproteins in NHLF (*n* = 4) after 143b exosome treatment across 39 analytes. **B,** Representative images of untreated and 143B-treated F62 and M48 NHLF phosphoprotein arrays. **C,** Analysis of MAPK pathway activity comparing untreated control and pooled OS exosome–treated (*n* = 5) F62 NHLF measured by bead-based phosphoprotein arrays. Two-way ANOVA and Sidak’s multiple comparisons test were used to analyze bead-based assays, * < 0.05. **D,** Endpoint fibroblast proliferation across three independent donors comparing untreated serum-free control and OS exosome (*n* = 4)–treated fibroblasts and corresponding representative live-cell imaging. Results show mean ± SD of technical replicates. Two-way ANOVA and Tukey’s multiple comparisons test were used. *, <0.05; **, <0.01; ****, <0.0001. **E,** Western blot analysis of lysates generated from untreated serum-free control and exosome (*n* = 4)-treated NHLF (*n* = 3) to determine the expression of canonical CAF markers α-SMA and fibroblast activation protein (FAP), followed by densitometry analysis.

Tumor exosome internalization has been shown to modulate recipient target cell phenotype through activation of a host of signal transduction pathways (36). As a first step to identify potential molecularly targetable pathways mediating OS exosome–induced the activation of LFs, we semiquantitatively analyzed phosphorylation changes in 46 different kinases across four separate donor LFs following 4 days of “education” with 143B OS exosomes (F62, M12, M48, and M54). Focusing only on those kinases that were detectable across all four donor LFs, 143B exosome treatment induced variably increased phosphorylation of seven different kinases relative to donor-matched untreated control fibroblasts, including HSP27, GSK-3β, PLC-γ1, STAT5A/B, WNK1, PRAS40, and STAT3 with significantly increased phosphorylation of HSP27, PRAS40, and STAT3 across all four donors (Fig. 3A and B).

As all three of these molecules represent either substrates or upstream activators of the MAPK and Akt/mTOR pathways, we next sought to analyze the activation of these specific pathways using a quantitative bead-based phosphoprotein assay. For these assays, we treated F62 donor LFs with exosomes derived from a larger panel of OS cell lines (*n* = 5; 143B, HOS, MG63.0, MG63.2, SAOS) and compared OS exosome–induced kinase activation in LFs with that observed for exosomes derived from a panel of five other highly lung metastatic, non-OS human tumor cell lines. Indeed, similar to results observed with the immunoblot kinase array, F62 NHLF relative phosphorylation levels across treatment with all *n* = 5 OS cells demonstrated significant increases in HSP27, p38, and ERK1/2 phosphorylation relative to NHLF treatment with exosomes derived from non-OS tumor cell lines (Fig. 3C); however, no statistically significant phosphoproteins were upregulated in the AKT/mTOR pathway (Supplementary Fig. S1). These data identify key intracellular signal transduction pathways in which activation in response to OS-derived exosomes is conserved across multiple OS cell lines and donor fibroblasts.

Given that OS exosome treatment of LFs induced (i) enrichment of gene signatures associated with cell proliferation and (ii) phosphorylation of pro-proliferation/survival signal transduction pathways that have been implicated in the transition of resident fibroblasts to a canonical CAF phenotype in primary tumors (37), we hypothesized that OS exosomes may be mediating a similar global transition in cell state from a quiescent LF to a CAF phenotype. To investigate this, NHLF survival and proliferation in response to OS exosome treatment were evaluated with live-cell imaging. Compared with untreated control NHLF, we observed statistically significant increases in NHLF survival/proliferation in response to treatment with HOS, MG63.0, and MG63.2 exosomes across three independent fibroblast donors (Fig. 3D). Additionally, we evaluated the expression of canonical CAF markers, α-SMA and fibroblast activation protein, by Western blot analysis of OS exosome–treated NHLFs; however, no significant upregulation of these markers in response to OS exosome treatment was observed (Fig. 3E).

### OS exosomes induce an inflammatory, protumorigenic secretome in primary human LFs

**Figure 4:**
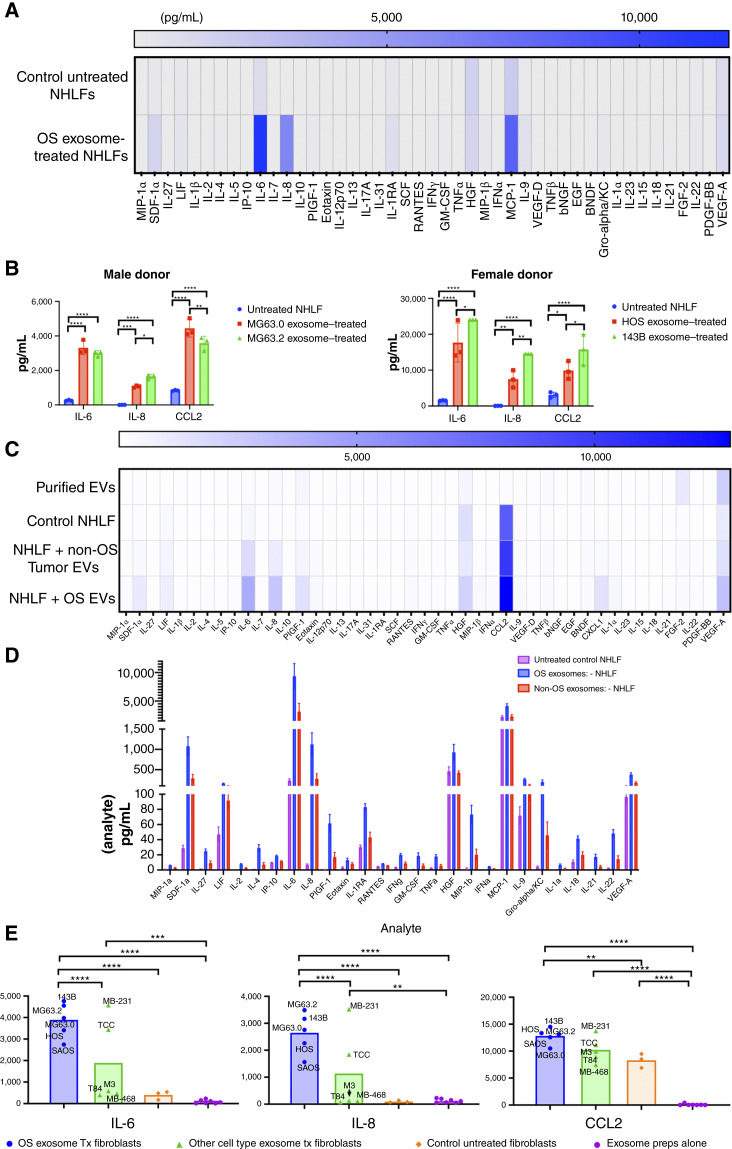
OS exosomes induce a protumorigenic secretory profile in NHLF. **A,** Heatmap depicting cytokine secretion in supernatants of pooled OS-educated (*n* = 4) NHLF (*n* = 2) assessed by a 45-plex, bead-based cytokine panel. Multiple *t* tests of male and female combined, mean values (pg/mL) ****, <0.0001. **B,** Significantly upregulated cytokines from **A**, IL-6, IL-8, and CCL2, with sex-matched NHLF donor to OS exosome cell line (male, MG63.0/MG63.2; female, 143b/HOS). Two-way ANOVA and Tukey’s multiple comparisons test were used. *, <0.05; **, <0.01; ***, <0.001; ****, <0.0001. **C,** Heatmap depicting cytokines in pooled exosome preparations alone from OS and other cell types (*n* = 10), supernatants of pooled untreated fibroblasts (*n* = 3), pooled exosome-treated NHLF from histotypes other than OS (*n* = 5), and OS exosome–treated (*n* = 5) NHLF (*n* = 3). **D,** Bar graph showing significant differential cytokine expression across 27 analytes when comparing NHFL (*n* = 3) treated with OS exosomes (*n* = 5) and other cell types (*n* = 5). **E,** Bar graphs depicting mean cytokine concentrations (pg/mL) from the three most significantly upregulated cytokines from **C**, IL-6, IL-8, and CCL2, and the respective cell lines’ cytokine concentrations. Two-way ANOVA and Tukey’s multiple comparisons test were used. *, <0.05; **, <0.01; ***, <0.001; ****, <0.0001.

CAFs within the primary TME have been shown to mediate their protumorigenic functions through the secretion of a variety of soluble mediators, including growth factors, cytokines, and chemokines. As our transcriptomic analysis of OS exosome–treated LFs suggested enrichment of a similar iCAF-like phenotype, we hypothesized that normal LFs would respond to priming with OS-derived exosomes in a similar manner, upregulating the secretion of known prometastatic cytokines. To characterize the secretory phenotype of NHLF in response to OS exosome “education,” CM from OS exosome–treated fibroblasts were assessed using a bead-based, 45-plex cytokine array. Analysis of pooled triplicate means from *n* = 2 NHLF donors (one male and one female) treated with OS exosomes derived from *n* = 4 cell lines (MG63.0, MG63.2, HOS, and 143B) demonstrated significantly (adjusted *P* < 0.05) increased secretion of IL-6, CXCL8, and CCL2 in the CM of OS exosome–“educated” LFs as compared with untreated NHLFs (Fig. 4A), with mean increases in OS exosome–educated versus control NHLFs of 10,876, 6,446, and 6,077 pg/mL for IL-6, CCL2, and CXCL8, respectively. Additionally, analysis of this data using NHLFs from both female (F62) and male (M12) donors sex-matched and paired to isogenic low- and high-metastatic respective male (MG63.0/MG63.2) and female (143B/HOS) OS cell lines demonstrated that although secretion of these molecules does not appear to be dependent on the sex of the OS exosome donor or recipient fibroblast cell line, there was significantly increased fibroblast secretion of IL-6, CCL2, and CXCL8 in response to exosomes derived from the high-metastatic 143B clone and increased CCL2 secretion in response to exosomes from the high-metastatic MG63.2 clone, as compared with the low-metastatic subclones (Fig. 4B).

Next, to determine if this OS exosome–induced secretory profile in NHLFs was a unique response to OS cells, given their high-metastatic tropism for the lung, or conserved across other highly metastatic tumor types, we also isolated exosomes from the same panel of five cell lines utilized in the phosphokinase arrays, representing a variety of other tumor histotypes that also display an enhanced proclivity for lung metastasis, including melanoma (Malme-3M), colorectal carcinoma (T84), urothelial carcinoma (TCC-Sup), and two triple-negative breast cancer cell lines (MB-231 and MB-468). Utilizing the same 45-plex bead-based cytokine array, analysis of supernatants from control untreated NHLFs (*n* = 3 independent donors) or NHLFs “educated” with exosomes derived from OS cells (*n* = 5) or tumor cells of other non-OS histotypes (*n* = 5) demonstrated significant (adjusted *P* < 0.05) differential upregulated secretion of 27 cytokines/chemokines/growth factors in OS exosome–primed LFs as compared with the priming of NHLFs with exosomes of non-OS tumor histotypes (Supplementary Table S5; Fig. 4C and D), with IL-6, CXCL8, and CCL2 again demonstrating the greatest mean difference between OS exosome–treated NHLFs versus non-OS exosome–treated NHLFs (Fig. 4D and E; Supplementary Table S5). Lastly, these same purified exosome preparations used for fibroblast education were also analyzed to determine whether these observations were the result of these cytokines being uniquely present within the exosomes themselves or possibly through their concentration during the exosome isolation process; however, minimal-to-no detectable levels of these cytokines were observed in the exosome preparations (Fig. 4C and E). Together, these data demonstrate that OS exosome treatment of primary human LFs *in vitro* significantly upregulates the secretion of known prometastatic cytokines and chemokines, including molecules previously demonstrated by us and others to be implicated in OS lung metastasis or MAF function (38). Moreover, these data also demonstrate that this effect is both conserved across multiple biologically independent fibroblast donors and is greater in response to OS exosomes compared with exosomes isolated from other highly lung metastatic tumor histotypes.

### Priming of NHLF with OS-derived exosomes enhances OS cell survival in a primary human LF–OS cell coculture model

**Figure 5:**
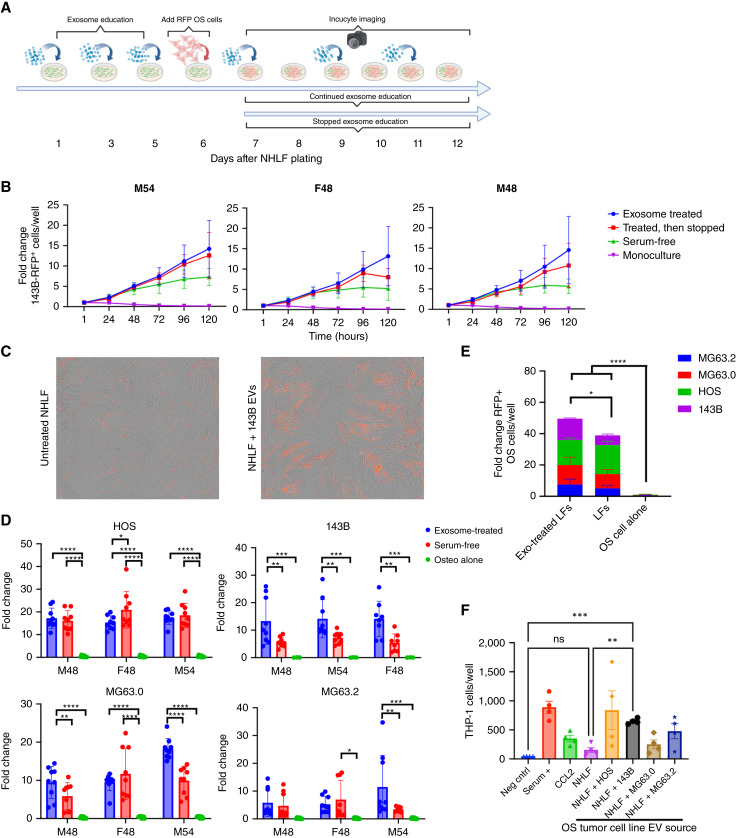
Exosome priming of NHLF promotes increased OS survival in an *in vitro* coculture model, and exosome-treated fibroblast supernatants promote monocyte migration. **A,** Schematic depicting the OS cell–NHLF coculture model. **B,** Fold change growth curves of RFP-labeled 143b OS cells in coculture with three independent NHLF donors that were treated with 143b exosomes throughout the experiment, only prior to OS cell seeding, serum-free untreated control, or OS cells in monoculture. **C,** Representative images of RFP-labeled 143b cells (red) in coculture with NHLF in untreated serum-free control or 143b exosome–treated conditions. Means of three biological replicates ± SD. **D,** Endpoint fold change from coculture experiments across three independent NHLF donors comparing exosome–treated, untreated serum-free control, and OS cells in monoculture from four OS cell lines. Means of three biological replicates ± SD. **E,** Pooled endpoint fold change comparing exosome–treated, untreated serum-free control, and OS cells in monoculture across three independent NHLF and four OS cell lines. **F,** Migration of THP-1 monocytes toward untreated fibroblast supernatants and OS exosome–treated (*n* = 4) supernatants. Two-way ANOVA and Tukey’s multiple comparisons test were used. *, <0.05; **, <0.01; ***, <0.001; ****, <0.0001.

Given that OS exosome treatment significantly changed the secretome of LFs, including the upregulation of cytokines known to have paracrine growth-promoting actions on tumor cells, we sought to determine the functional impact of OS exosome–primed NHLF on OS cell proliferation and survival. To do this, we designed an NHLF–OS cell coculture assay to mimic early tumor cell dissemination to the lung. NHLFs were either pretreated or continuously primed with OS-derived exosomes before and after low-density seeding (100 cells/well) of matched RFP + OS cell lines in serum-free media onto a monolayer of exosome-primed NHLF, onto untreated NHLF, or in monoculture control wells (Fig. 5A). Growth curves comparing the fold change of 143B RFP cell growth/survival over time within the four treatment conditions demonstrate significantly enhanced growth within exosome-primed NHLFs across three donors compared with unprimed NHLFs or OS cells in monoculture (Fig. 5B). Importantly, this growth advantage occurred whether NHLFs were treated with exosomes prior to OS cell seeding only or continuously throughout the duration of culture and was not observed for OS exosome treatment of OS cell monocultures, suggesting that OS exosomes themselves have no direct effect on OS cell proliferation, either in the presence or absence of LFs (Fig. 5B; Supplementary Fig. S2). Representative images show the growth advantage conferred to 143B RFP cells by OS exosome–primed NHLFs compared with NHLFs that did not receive exosome treatments (Fig. 5C). Moreover, OS cell survival was significantly enhanced across multiple NHLF donors and OS cell lines when LFs were primed with exosomes from the respective OS cell line. Exosome priming of all three NHLF donors enhanced 143B growth/survival, whereas two NHLF donors (M48 and M54) enhanced MG63.0 cell survival/proliferation and one NHLF donor (M54) significantly increased MG63.2 cell survival/proliferation (Fig. 5D and E).

As OS exosome treatment of LFs promotes significant secretion of CCL2, a chemokine implicated in the recruitment of inflammatory monocytes to early metastatic sites, we sought to determine the functional impact of OS exosome–induced NHLF CCL2 production on the chemotactic potential of human THP-1 monocytes. We observed significantly increased THP-1 migration to OS exosome–treated NHLF supernatants, with significant migration in 143B–treated NHLF supernatants compared with the negative control and untreated fibroblast supernatants (Fig. 5F).

## Discussion

In 1889, Stephen Paget first postulated his “seed and soil” hypothesis, seeking to explain why certain cancers are predisposed to metastasize to specific distant organs, inciting research into the nonrandom pattern of cancer metastasis. Over a century later, research investigating the extrinsic factors of cancer cells that contribute to metastatic dissemination has identified small, tumor-derived extracellular vesicles as important mediators of this process in multiple cancers with distinct metastatic organ predilections, such as melanoma, pancreatic, and breast cancers (39). However, despite OS’s striking predilection for lung metastasis, there have been limited investigations into OS tumor–derived extracellular vesicles and their ability to interact with nonmalignant resident cell types of the lung. In this study, we demonstrate that normal LFs rapidly and efficiently internalize OS exosomes across a panel of OS cell lines and fibroblast donors. This OS exosome priming induces distinct transcriptomic changes in LFs, including upregulation of genes and pathways of canonical CAF phenotypes, including both myofibroblastic (myCAF) and inflammatory (iCAF) subsets. Moreover, we provide *in vitro* evidence of OS exosomes’ ability to drive normal LFs toward protumorigenic CAF functions, including OS exosome–mediated fibroblast proliferation, induction of fibroblast secretion of key cytokines and chemokines previously implicated in OS lung metastasis progression, and promotion of OS cell survival in a fibroblast–OS cell coculture model. These data suggest that OS exosomes can promote the transition of normal LFs into MAF phenotypes with protumorigenic functional consequences, providing a rationale for future preclinical investigations testing genomic or pharmacologic approaches to interrupt this intercellular signaling as potential antimetastatic strategies for OS.

Although the uptake of exosomes from all six OS cell lines occurred in more than 90% of LFs and across all four donor fibroblast cell lines, the degree to which uptake occurred was not equal, both in terms of OS cell line source exosomes and between LF donors. This difference in uptake may simply be the result of inherent biological variability across human donors or a more specific lock-and-key mechanism based on unique exosome and cell-surface ligand–receptor interactions. Indeed, prior studies have demonstrated a dependency on specific cognate integrin receptor repertoires between exosome and target cells in directing organ-specific homing (24, 40–44); however, these mechanisms have not been described specifically for LFs. Although the molecular machinery mediating OS exosome uptake by LFs was not specifically evaluated in this study, we did identify MAPK/ERK pathway activation in LFs in response to OS exosome priming. Importantly, signaling components of the MAPK pathway have been previously reported to mediate the uptake of breast cancer EVs by LFs. In a high-content screen of FDA-approved anticancer drugs, Wan and colleagues (45) identified MEK2-dependent micropinocytosis as a requirement for LF uptake of MDA-MB-231 breast cancer EVs.

In addition to MAPK/ERK pathway activation, kinase profiling of LFs in response to OS exosome priming also identified significant activation of p38/MAPK and the upstream activator of ERK1/2, STAT3. ERK1/2, p38 MAPK, and STAT3 signaling cascades have all been shown to mediate the transition from a quiescent fibroblast phenotype to a characteristic CAF phenotype associated with CAF-induced cancer progression, chemoresistance, and immune suppression (46–50). Notably, STAT3 has been shown to be overexpressed in tumor tissues of patients with OS, and high STAT3 protein levels were associated with increased metastasis and reduced disease-free and overall survival. However, whether STAT3 overexpression in these tissues of patients with OS was purely restricted to the tumor compartment or was also a result of CAF contribution remains unclear, as coimmunolabeling for STAT3 and tumor- or fibroblast-specific cell phenotype markers was not performed in this study (51).

These protein kinases we identified being activated in OS EV–primed LFs are also known drivers of the secretion of key tumor-promoting cytokines and chemokines such as IL-6, IL-8, and CCL2 across a range of cell types, including fibroblasts (52, 53). In line with these observations, of the 45 cytokines, chemokines, and growth factors assessed in the supernatants of OS exosome–treated fibroblasts, IL-6, CXCL8, and CCL2 were the most significantly upregulated proteins, all canonical CAF-secreted cytokines (54, 55). In the context of PMN formation, IL-6 can elicit both direct intrinsic effects on cancer cells, such as cell-cycle progression, invasion, and metastasis, and indirect, cell-extrinsic effects on the TME, including the promotion of angiogenesis, tumor-promoting inflammation, and immune suppression (56). CXCL8 is a pleiotropic signaling molecule known to mediate CAF promotion of angiogenesis, EMT, and the recruitment of myeloid-derived suppressor cells and tumor-associated neutrophils (57, 58). Specifically, in the context of OS, IL-6, CXCL8, and CCL2 have all been linked to the promotion of OS lung metastatic progression. Gross and colleagues (38) demonstrated that both IL-6 and CXCL8 secretion by OS cell lines correlated strongly with lung colonization efficiency in murine xenograft models and that the combined blockade of these molecules prevented metastatic growth. Moreover, they demonstrated that both IL-6 and CXCL8 mRNA and protein expression were significantly upregulated in human lung metastases of patients with OS as compared with matched primary tumors. Intriguingly, IL-6 and CXCL8 immunolabeling was localized along the tumor–lung interface of these metastases, a microanatomic region often spatially enriched for CAFs, suggesting that LFs may be contributing to this IL-6 and CXCL8 expression, given that dual labeling for tumor- or fibroblast cell–specific phenotype markers was also not performed in this study.

Similarly, targeting the CCL2-CCR2 signaling axis in OS has emerged as a potential avenue for antimetastatic immunotherapy. Prior work has demonstrated that both human and canine OS cell lines overexpress CCL2, which is correlated with increased monocyte migration *in vitro* and monocyte enrichment in patient lung metastases. These studies also showed that losartan, an angiotensin II receptor antagonist used in the treatment of hypertension, can block *in vitro* CCL2- and OS cell–elicited monocyte recruitment through noncompetitive inhibition of CCL2-induced ERK1/2 activation, independent of angiotensin II receptor signaling. In multiple preclinical mouse models, losartan suppressed lung metastasis via blockade of CCL2-CCR2 monocyte recruitment, and in a clinical trial in dogs with spontaneous lung metastatic OS, losartan in combination with the multi-kinase inhibitor toceranib was associated with modulation of CCL2-CCR2 pharmacodynamic endpoints and a clinical benefit rate of 50% (59, 60). As a result of this promising preclinical data, a clinical trial evaluating losartan in combination with sunitinib as an antimetastatic therapy in human pediatric and adult patients with relapsed or refractory OS is currently underway (ClinicalTrials.gov, NCT03900793). Thus, our data suggest that LF targeting within the OS TME may represent another potential strategy to block CCL2-mediated monocyte recruitment to lung metastases.

Recent advances in single-cell multiomic technologies have shed light on the spatial, transcriptomic, and functional heterogeneity of CAF subpopulations. CAFs have been subclassified into at least three highly plastic subtypes that share several common characteristics while diverging in others. These subtypes have been described as myofibroblast, antigen-presenting, and inflammatory CAFs or myCAF, apCAF, and iCAF respectively (32). Our transcriptomic profiling of LFs in response to OS exosome education revealed nearly 500 significant differentially expressed genes, including many associated with cytokine/chemokine expression, interferon expression, and complement, all genes typical of the iCAF phenotype. Moreover, genes associated with the myCAF phenotype that regulate ECM remodeling, such as various types of collagens, matrix metalloproteases, elastin, and periostin, were also highly upregulated upon OS exosome treatment. Although fibroblast-mediated remodeling or secretion of ECM components within metastatic sites is known to promote circulating tumor cell seeding and outgrowth, the specific upregulation of periostin by LFs in response to OS EVs is intriguing. Specifically, tumor cell–induced expression of periostin by resident fibroblasts has been shown to be essential for breast cancer lung metastasis through periostin-mediated enrichment of Wnt ligands, and Wnt receptor expression in OS has been associated with OS metastasis in rodent models and worse event–free survival in patients (61, 62). In contrast, genes typically associated with the apCAF phenotype, such as serum amyloid A3, secretory leukocyte peptidase, and major histocompatibility complex 2, were not differentially expressed in response to OS exosome education (63). In addition to the upregulation of genes that partition CAFs into their respective subtypes, pathway analysis revealed gene sets that promote other hallmark CAF functions, including angiogenesis, adhesion, and proliferation.

These results, when viewed through the lens of the CAF subtype framework, suggest that OS-derived exosomes may have the capacity to promote the transition of normal lung-resident fibroblasts toward MAFs exhibiting both myCAF and iCAF phenotypes, providing a rationale for future *in vivo* studies evaluating this mechanism within the context of OS lung PMN formation. These findings parallel those of two other studies evaluating the molecular and functional changes of fibroblastic cell types in response to human OS cell–derived EV treatment *in vitro*. Adipose-derived mesenchymal stem cells (MSC) were also observed to efficiently internalize 143B OS cell EVs, which induced MSC proliferation and IL-6 secretion (64), just like the inflammatory and proliferative phenotypes we observed in our EV-educated primary LFs. Similarly, the investigation of the effects of human OS cell–derived EVs on fetal LF cell lines WI-38 and MRC-5 demonstrated that 143B OS-derived EVs drive myCAF differentiation of normal fetal LFs, characterized by increased invasion, α-SMA expression, and fibronectin production (65), analogous to the myCAF gene signature enrichment we observed in our RNA sequencing analysis of OS EV–educated LFs.

Although these various CAF subtypes are associated with defined mechanisms of tumor promotion in other cancer types, limited evidence about the role of CAFs in mediating protumorigenic functional responses in OS cells, such as changes in cell survival or proliferation, exists. Given that OS exosome priming of LFs induced many of the phenotypes seen in previously described CAF subtypes, we sought to leverage this response to develop a simplistic and scalable *in vitro* coculture model that attempts to recapitulate the early lung metastatic microenvironment and the impact of OS cell–derived LF cross-talk on OS cell survival. Under conditions of cellular stress induced by serum deprivation and low seeding density, OS cells were fully dependent on LFs to sustain their proliferation. Although OS cells can typically survive under serum-starved conditions when plated at higher densities, OS cells seeded at low densities, like those used as controls, have difficulty attaching to the well bottom, and after 24 hours, they appear to bleb, begin to die, and fully lift off the plastic of the well. The same OS cells seeded in wells that contain fibroblasts quickly embed themselves within the fibroblast monolayer and begin to proliferate. Proliferation of OS cells embedded within the fibroblast monolayer was significantly more pronounced upon OS exosome activation of these LFs and was characterized by multifocal, clustered outgrowths indicative of OS lung metastasis, as demonstrated in Fig. 5C. Interestingly, two- to threefold higher OS cell proliferation occurred whether fibroblasts were primed by exosomes prior to OS cell seeding alone or exosome education continued after seeding throughout the remainder of the experiment, compared with “uneducated” fibroblasts in the serum-free condition, in which OS cell proliferation leveled off, as demonstrated in Fig. 5B. This suggests that exosome priming is sufficient to induce phenotypic changes in fibroblasts that are conducive to enhanced OS cell proliferation, and continued exosome education is not required to observe this effect. However, the mechanism by which OS EV–primed LFs promote the enhanced OS cell growth advantage observed in this model remains to be defined, whether that be ECM outside-in signaling, cytokine-mediated paracrine effects, or juxtacrine signaling through cell-to-cell contact with exosome–primed fibroblasts. To our knowledge, only two other studies have evaluated the functional effects of OS EV–primed fibroblastic cell interactions with OS cells, which demonstrated that human OS EV–primed MSCs can promote OS primary tumor growth, metastasis, and drug resistance in murine xenograft models (64, 66). These studies demonstrated that EV-associated TGFβ induced IL-6 secretion in MSCs, which drove tumor progression through paracrine STAT3 activation in OS cells, suggesting that the increased IL-6 secretion we observed in response to OS EV treatment of primary LFs may be mediating this enhancement of OS cell survival and proliferation in our model. Thus, our data also support the notion that exosome priming and reprogramming of LFs may be a critical first step in reorganizing the lung microenvironment in preparation for supporting circulating OS cell seeding and outgrowth and warrant future *in vivo* investigation of these phenomena.

In conclusion, we demonstrate that OS-derived exosomes have significant functional impacts on normal LFs, inducing a rapid transition in phenotype following exosome uptake, including distinct kinase activation, increased fibroblast proliferation, and enhanced secretion of known prometastatic cytokines and chemokines previously implicated in OS lung metastasis. Transcriptomic profiling of OS exosome–primed LFs confirmed significant enrichment of canonical myofibroblastic and inflammatory CAF gene signatures. Importantly, this OS exosome–mediated transition of LFs into CAFs was associated with increased monocyte recruitment and promotion of OS cell survival and proliferation under conditions of cellular stress *in vitro*. Collectively, these data suggest that an OS exosome–LF signaling circuit may represent an important event in orchestrating the establishment of a metastasis-permissive microenvironment in the lung. Thus, these data provide a foundation for future *in vivo* studies investigating these mechanisms within preclinical murine models of OS metastasis to determine whether this intercellular communication network may represent a novel therapeutic target for interrupting the premetastatic microenvironment in OS.

## Supplementary Material

Figure S1 legend Figure legend for Akt Phospho-protein assay in osteosarcoma exosome treated lung fibroblasts.

Figure S1 Bar graph quantifying relative phospho-protein activity in the Akt signaling pathway for control vs. exosome treated lung fibroblasts

Supplementary Table S1 List of fibroblast donor by age, sex, and lot number

Supplementary Table S2 Uptake of labeled exosomes by OS exosome donor cell line and recipient fibroblast cell line by percent

Supplementary Table S3 List of differentially expressed genes among OS exosome treated lung fibroblasts

Supplementary Table S4 Enriched pathways of gene sets among OS exosome treated lung fibroblasts

Supplementary Table S5 Raw cytokine/chemokine data from OS exosome treated fibroblasts assessed by bead-based multiplex panel
