# Supplementary material for: Osteosarcoma Exosome Priming of Primary Human Lung Fibroblasts Induces an Immune Modulatory and Protumorigenic Phenotype
Source: Cancer Res Commun. 2025 Apr 11;5(4):594–608. doi: 10.1158/2767-9764.CRC-24-0371 (PMC11987067; doi:10.1158/2767-9764.CRC-24-0371)
Supplement: Figure S1 legend — Figure legend for Akt Phospho-protein assay in osteosarcoma exosome treated lung fibroblasts. [file crc-24-0371_figure_s1_legend_suppsfl1.docx]

**Fig. S1**. Analysis of Akt pathway activity comparing untreated control and pooled, OS-exosome treated (n=5) F62 NHLF measured by bead-based phospho-protein array. No significant differences in phospho-protein levels were detected between groups based on 2-way ANOVA and Sidak’s multiple comparisons test.
