# Supplementary material for: Osteosarcoma Exosome Priming of Primary Human Lung Fibroblasts Induces an Immune Modulatory and Protumorigenic Phenotype
Source: Cancer Res Commun. 2025 Apr 11;5(4):594–608. doi: 10.1158/2767-9764.CRC-24-0371 (PMC11987067; doi:10.1158/2767-9764.CRC-24-0371)
Supplement: Figure S1 — Bar graph quantifying relative phospho-protein activity in the Akt signaling pathway for control vs. exosome treated lung fibroblasts [file crc-24-0371_figure_s1_suppsf1.pdf]

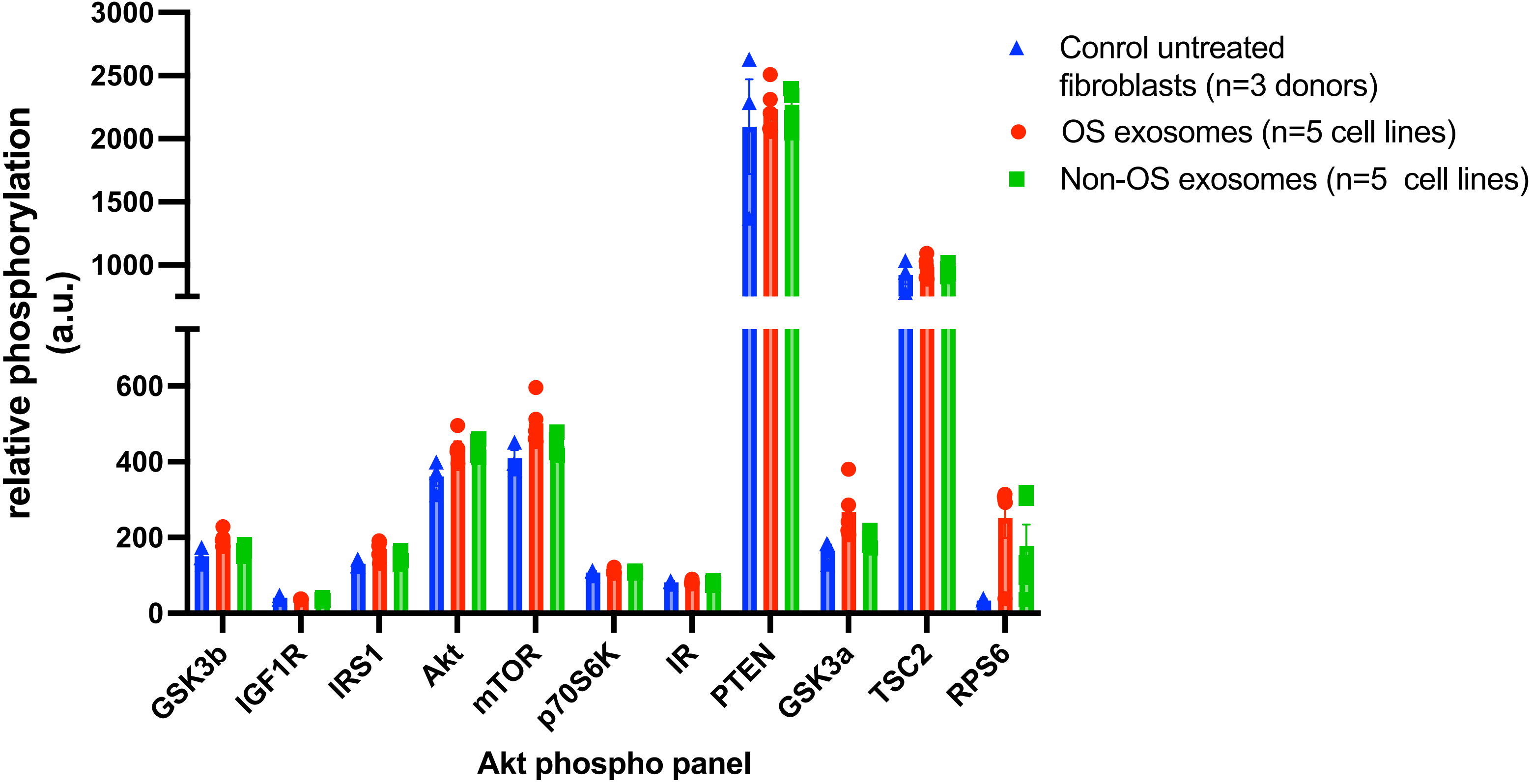

3000
2500
2000
1500
1000
600
400
200
0
relative phosphorylation (a.u.)
GSK3b
IGF1R
IRS1
Akt
mTOR
p70S6K
IR
PTEN
GSK3a
TSC2
RPS6
Akt phospho panel
Conrol untreated fibroblasts (n=3 donors)
OS exosomes (n=5 cell lines)
Non-OS exosomes (n=5 cell lines)
